# *De novo* transcriptome analysis of the excretory tubules of *Carausius morosus* (Phasmatodea) and possible functions of the midgut ‘appendices’

**DOI:** 10.1371/journal.pone.0174984

**Published:** 2017-04-06

**Authors:** Matan Shelomi

**Affiliations:** 1 Department of Entomology, National Taiwan University, Taipei, Taiwan; 2 Department of Entomology, Max Planck Institute for Chemical Ecology, Jena, Thüringen, Germany; Institute of Zoology Chinese Academy of Sciences, CHINA

## Abstract

The Malpighian tubules are the insect excretory organs, responsible for ion and water homeostasis and elimination of nitrogenous wastes. Post-genomic assays suggest they also metabolize and detoxify xenobiotic compounds and have antimicrobial properties. The Phasmatodea have an additional, unique set of excretory organs referred to predominantly as midgut appendices. Their function and how it compares to phasmid and other insect Malpighian tubules is unknown. Hypotheses include carbonic anhydrase activity, calcium and metal cation sequestration, and xenobiotic transport. This work presents the first comparative transcriptomic analysis of the Phasmatodean excretory organs, using the model insect *Carausius morosus*. I produced *de novo* transcriptomes of the midgut appendices, midgut wall, and Malpighian tubules, and looked for differentially expressed genes associated with putative organ functions. The appendices differentially and highly express lipid transport and metabolism proteins, and the biomineralization gene otopetrin. The Malpighian tubules differentially and highly express acid phosphatases and multiple transporter types, while appendices express fat-soluble vitamin and peptide transporters. Many defense proteins such as multidrug resistance proteins, ABC transporters, cytochrome P450’s, and glutathione-S-transferases were differentially expressed in specific excretory organs. I hypothesize that the appendices and Malpighian tubules both have defensive / xenobiotic metabolism functions, but each likely target different substrates. Phasmid Malpighian tubules excrete as in other insects, while the appendices may predominantly regulate amino acids, fats, and fat-soluble compounds. Lipid metabolism in insects is poorly understood, and the Phasmatodea may thus serve as a model for studying this further.

## Introduction

The main organs of insect excretion, analogous to the human nephrons, are the Malpighian tubules, which usually arise at the junction of the midgut and hindgut. Not only do they eliminate nitrogenous wastes and regulate water/ion balances in the hemolymph, but also they function in the elimination and metabolism of xenobiotics such as plant secondary toxins [1]. Pioneering work on the excretory physiology of Malpighian tubules was done by J.A. Ramsay in the 1950’s, in which he perfected techniques of collecting urine directly from a single tubule dissected and placed in a drop of saline under liquid paraffin, using the model insects *Rhodnius* [2] and the laboratory or Indian walking stick, *Carausius morosus* (previously *Dixippus morosus*) [3–6]. In the 1970’s, Taylor performed a series of physiological and ultrastructural studies on the Malpighian tubules, again using *Carausius* [7–10]. Tracking the fate of injected dyes or alkaloids as they are absorbed by the tubules for elimination is another technique with considerable history [11–12], which confirmed that the tubules excrete such xenobiotic solutes via active transport [13–16]. More recently, electrophysiological [17] and post-genomics era assays, primarily on model insects *Drosophila* and *Manduca*, have identified the proteins associated with the various transport functions of the tubules, namely V-ATPase proton pumps working in concert with cation/proton antiporters, demonstrating that the tubules can actively transport solutes [18–21]. They also revealed a large complement of defense proteins such as multidrug resistance proteins (a class of ATP-binding cassette, or ABC, transporters) [22], NO synthase (used in immune sensing) [23], Diptericin (an antimicrobial peptide effective against gram-negative bacteria whose expression is upregulated by NO synthase) [24], and xenobiotic metabolism/detoxification/conjugation genes such as cytochrome P450s, glutathione-S-transferases and alcohol dehydrogenases [24–26]. As many of these genes have human homologues involved in kidney disorders, the Malpighian tubules have been declared a model for human renal disease [21, 27].

**Fig 1:**
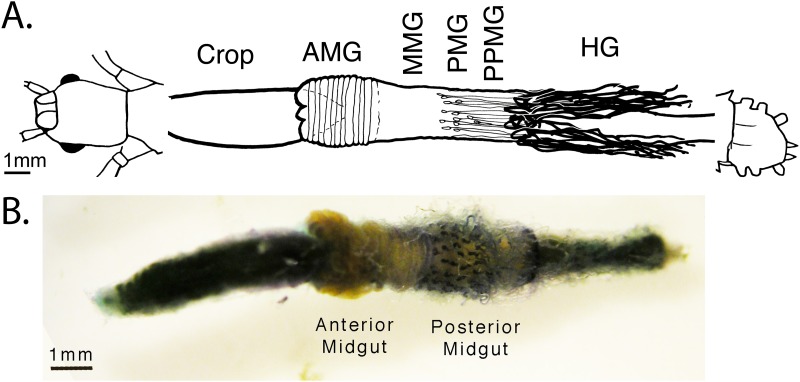
Digestive and excretory system of the Phasmatodea. A) Schematic and B) dissection of the alimentary canal from *Aretaon asperrimus*, (Heteropterygidae) typical of other Phasmatodea [32]. The insect was vitally stained with New Methylene Blue N and dissected 6 days later. The appendices [violet] appear on the posterior midgut. The Malpighian tubules [colorless] originate at the midgut/hindgut junction, trailing over the posterior midgut before going towards the posterior end of the insect. The gut section between the two, the “post-posterior midgut,” was used for our midgut wall (MGWall) samples, excluding any tubules. Key: AMG = anterior midgut. HG = hindgut, MMG = middle midgut. PMG = posterior midgut. PPMG = post-posterior midgut. The schematic is reused with permission from this author’s previously published work [32].

In addition to the Malpighian tubules, some types of excretion are performed as ancillary functions of other organs such as the midgut [28] or pericardial tissue [29]. However, a separate and unique excretory organ system evolved in the Phasmatodea, including *Carausius*. They have been given many names, but are referred to mostly as “appendices of the midgut” or some translation thereof [30–36]. They consist of long, coiled tubules approximately 1/3 the diameter of Malpighian tubules that end blindly in the hemolymph near the anal end of the insect, and are proximally connected to pear-shaped ampullae 300–500μm long that project from and open into the posterior end of the midgut (Fig 1, S1 Fig). The tubules are highly motile, well-tracheated [32] with muscular elements spiraling across their lengths as in Malpighian tubules [36] and showing simple harmonic motion. The ampullae often appear filled with a yellow fluid, but neither they nor the tubules contain microbial symbionts [30]. An autapomorphy of the Phasmatodea [31], these “appendices” are not, as their name would suggest, vestigial, nor are they a displaced variant of the Malpighian tubules, though the two are likely homologous in origin. Evidence with vital staining confirms that the appendices actively transport solutes. However they do not transport the same stains as the Malpighian tubules, such as indigo carmine or methyl green [14, 28], nor stains associated with pericardial tissue, such as ammonium carmine [12, 29]. Instead they absorb and eliminate into the midgut lumen a unique set of predominantly cationic but otherwise chemically disparate stains, suggesting that, though excretory, their exact function has no known analogue in arthropod excretory tissue [32]. The only other clue to their function is an assay by Monteiro et al. [33] finding the highest specific activity of the alkalinizing enzyme carbonic anhydrase in the “Midgut protuberances,” which corresponds with the known alkalinity of the posterior midgut at and following the origin of the midgut tubules [30].

Thus, despite their having been described over a century ago [34, 35], we still know little about what the appendices actually do. Ramsey himself never successfully used his urine-collecting methods on the appendices despite having developed them on the Phasmatodea, or at least never published the results of his efforts. This is likely due to the appendices’ degradation in Ringer and other medias [36], and the difficulty of performing Ramsay assays on tubules with too narrow a lumen [19, 37]. Several authors studying Phasmatodea Malpighian tubules made no mention of the appendices altogether, including Taylor in his work on *Carausius* [7–10]. The extant hypotheses for the appendices’ function is excretion of solutes the Malpighian tubules cannot process, which could include xenobiotics, as well as carbonic anhydrase activity. Shelomi & Kimsey [32] predicted the appendices function in calcium regulation and/or organic alkaloid sequestration, while rejecting functions known for other insect midgut outcroppings such as microbial crypts [30]. Next-generation sequencing technologies such as transcriptomics can rapidly test these hypotheses and develop new insights into the function of these enigmatic organs, just as they revealed hitherto unknown or only hypothesized functions in the Malpighian tubules [24, 27]. Tissue-specific analyses can also identify tissue-specific functions and differentially expressed genes, including tightly expressed genes that could otherwise be overlooked in whole-organism tests [21, 26].

In this experiment, I returned to the model organism *Carausius morosus*, and generated *de novo* transcriptomes for three tissue types: the Malpighian Tubules (MpgT), appendices of the midgut (AoM), and the midgut wall itself (MGwall). Using RNA-Seq, I identified the highest and/or most differentially expressed genes in each tissue type, with the goal of determining what the function of the AoMs is on a molecular level.

## Results

### De novo transcriptome assembly and assessment

A total of 9 *C*. *morosus* cDNA libraries—three each from AoMs, MpgTs, and MGwalls—were produced from adult, female [the species is mostly parthenogenetic] insects. Illumina sequencing and processing generated 20 million reads (4.00 gigabases) of data per library, available in the Sequence Read Archive (SRA) of NCBI [see data availability statement]. The transcriptome assembly from the quality tested, trimmed, and pooled data produ was refined via duplications testing and fusion of contiguous sequences with the program CAP3 [38] to 73,143 non-redundant contigs (Tables 1 and 2). All contigs are either named “Contig#” [post-CAP3 contigs] or “Carausius_C#” [unchanged from original assembly]. The species most commonly represented in BLAST hits for the assembly as determined via Blast2GO v3.0.11 [39] were all animals and almost all insects, with the majority of identified hits belonging to the termite *Zootermopsis nevadensis* (S2 Fig), as expected given the close relationship of Phasmatodea with Blattodea/Isoptera relative to the other insects on the list [31].

**Table 1:** Transcriptome assembly contig length measurements.

	with scaffolded regions	without scaffolded regions
N75	599	468
N50	1206	932
N25	2322	1796
Minimum	250	50
Maximum	41,521	41,521
Average	863	662
Count	85,006	108,805

**Table 2:** Transcriptome assembly summary statistics.

	Count	Average Length	Total Bases
Reads	161,455,318	94.56	15,267,714,112
Matched	126,641,493	94.54	11,972,103,583
Not matched	34,813,825	94.66	3,295,610,529
Contigs	85,006	863	73,393,572
Reads in Pairs	69,329,932	326.56	
Broken paired reads	57,311,561	94	

### RNA-Seq and differential expression

**Fig 2:**
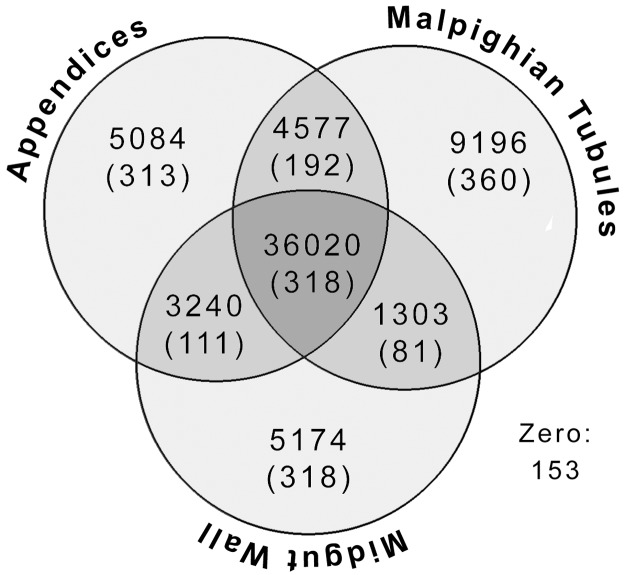
Venn diagram showing the distribution of transcripts in the tissue libraries. The number of sequences differentially expressed (and, in parentheses, the number highly expressed) in the midgut “appendices,” Malpighian tubules, the midgut wall, and pairs of these tissues are given. The center shows the number of sequences equally expressed in all three tissues (meaning no differential expression), with the number highly expressed in all three tissues in parentheses. “Zero” indicates the 153 reads that Cufflinks could not map.

Mapping the original library reads to the contigs from the CAP3 transcriptome using Cufflinks v2.2.1 resulted in 64,747 contigs with mapped reads. Cufflinks then identified 9196 contigs differentially expressed most highly in MpgTs relative to the other two tissues, 3240 such contigs in the MGWall, and 5084 in the AoMs (Fig 2). It also found 4577 contigs differentially expressed in both excretory organs (AoM + MpgT) relative to the midgut wall, 3240 to the midgut tissues (AoM + MGWall), and 1303 to the MGWall and MpgT. By defining high expression as any RPKM (reads per kilo base per million mapped reads) value ten times above the mean, I found 881 contigs highly expressed in the MpgTs, 924 in the AoMs, and 758 in the MGwall, including those highly expressed in more than one tissue type (318 highly expressed in all three). Combining these statistics I found 379 contigs highly and differentially expressed in the MpgT alone, 361 in the AoMs, and 427 in the midgut (Fig 2). From these, 126, 131 and 109 were unidentifiable by BLASTx searches.

Among the differentially and highly expressed genes in the appendices were multiple fatty-acid- and lipid storage droplet surface binding proteins, triaglycerol lipases, V-type proton ATPases, a retinol (Vitamin A) dehydrogenase, several alpha tocopherol (Vitamin E) transfer proteins, two glutathione-S-transferases, many cytochrome p450s, an otopetrin, beta-actins, and various proteases. Among the differentially and highly expressed genes in the Malpighian tubules were annexins, many cytochrome p450s and multidrug resistance proteins, prostatic acid phosphatases, many solute carrier transporter family members, and other transporters of calcium, protons, sodium, phosphates, monocarboxylates, anions, cations, trehalose, and vesicular glutamate. Among the differentially and highly expressed genes in the midgut wall were a great many ribosomal proteins, an alpha tocopherol transfer protein, triaglycerol lipases, peritrophin, ferritin, many antennal esterases, some cytochrome P450s, and many digestive proteins such as beta-galactosidase, glucosylceramidase, cellulase, maltase, polygalacturonase (pectinase), and various proteases (Table 3, S1 Table).

**Table 3:** Top identified, over-expressed transcripts of the *Carausius* appendices of the midgut.

Contig Name	Description [Blast2GO]	Hit Accession #	e-value	sim mean	length (bp)	mean AoM RPKM	log 2 fold change (vs MpgT)	log 2 fold change (vs MGwall)
Carausius_C45	Chymotrypsin BI	KDR14900.1	2.22E-59	0.65	611	7396.6	-8.86	-1.16
Carausius_C67	zinc metalloproteinase	AGM32350.1	4.13E-61	0.6	901	5579.6	-6.72	-8.86
Carausius_C392	lipocalin cytosolic fatty-acid binding	AGM32122.1	3.28E-62	0.82	680	3895.0	-3.57	-2.32
Contig52	Lipase member H	KDR14939.1	4E-42	0.55	1919	3860.6	-8.93	-1.19
Carausius_C126	Zinc metalloproteinase nas-14	KDR19395.1	8.8E-60	0.7	599	3595.2	-1.56	-8.27
Contig51	Glutathione S-transferase omega-1	KDR22870.1	6.22E-17	0.51	859	2743.4	-4.45	-1.23
Carausius_C197	lipocalin cytosolic fatty-acid binding	AGM32122.1	2.95E-61	0.82	973	2669.2	-3.65	-2.11
Carausius_C912	Triaglycerol lipase	KDQ97822.1	9.08E-45	0.55	1361	2631.0	-8.89	-1.17
Carausius_C444	V-type proton ATPase subunit partial	KDR08717.1	0	0.94	1573	2448.7	-0.82	-1.43
Carausius_C198	lipocalin cytosolic fatty-acid binding	AGM32122.1	4.3E-59	0.81	1005	2346.3	-3.37	-2.87
Carausius_C103	beta actin-5C	NP_511052.1	4.47E-124	0.99	653	2310.8	-0.94	-1.10
Carausius_C1664	zinc metalloproteinase	AGM32350.1	3.33E-25	0.61	483	2225.6	-6.15	-8.70
Contig348	Natterin-3 [kininogenase]	KDR19911.1	5.07E-48	0.66	1041	1933.0	-1.88	-2.74
Contig49	beta actin, partial	ADZ52965.1	4.00E-176	0.92	542	1608.6	-0.78	-0.88
Contig245	Zinc carboxypeptidase A 1	KDR22871.1	6.11E-93	0.71	2140	1495.5	-4.64	-4.48
Carausius_C1260	vacuolar-type H+-ATPase	AGO46410.1	0	0.98	3456	1286.8	-0.66	-1.24
Carausius_C1234	zinc metalloproteinase	AGM32350.1	2.08E-61	0.71	531	1212.7	-1.88	-8.14
Carausius_C395	Glutathione S-transferase omega-1	KDR22870.1	1.29E-19	0.5	1197	1124.9	-4.05	-1.81
Contig292	Na-dependent nutrient amino acid transporter	KDR22766.1	0	0.78	1519	1122.5	-7.38	-8.80
Carausius_C27	CD63 antigen	KDR11258.1	1.16E-27	0.64	1709	1049.6	-2.70	-4.11
Contig235	Retinol dehydrogenase 11	KDR07942.1	2.15E-93	0.75	999	1032.2	-3.91	-2.85
Carausius_C1903	Cytochrome P450 6k1	KDR14071.1	1.17E-27	0.74	346	950.1	-3.15	-2.24
Contig356	juvenile hormone esterase Est1	ACT53736.1	2.45E-69	0.6	898	941.0	-4.12	-3.01
Carausius_C25	CD63 antigen	KDR11258.1	4.67E-31	0.65	1665	922.6	-2.53	-4.06
Contig209	Cytochrome P450 partial	KDQ77054.1	2.56E-114	0.65	1195	907.1	-2.52	-1.43

Only genes that were both highly expressed [RPKM values >10x the mean] and differentially expressed in the appendices alone are included.

**Fig 3:**
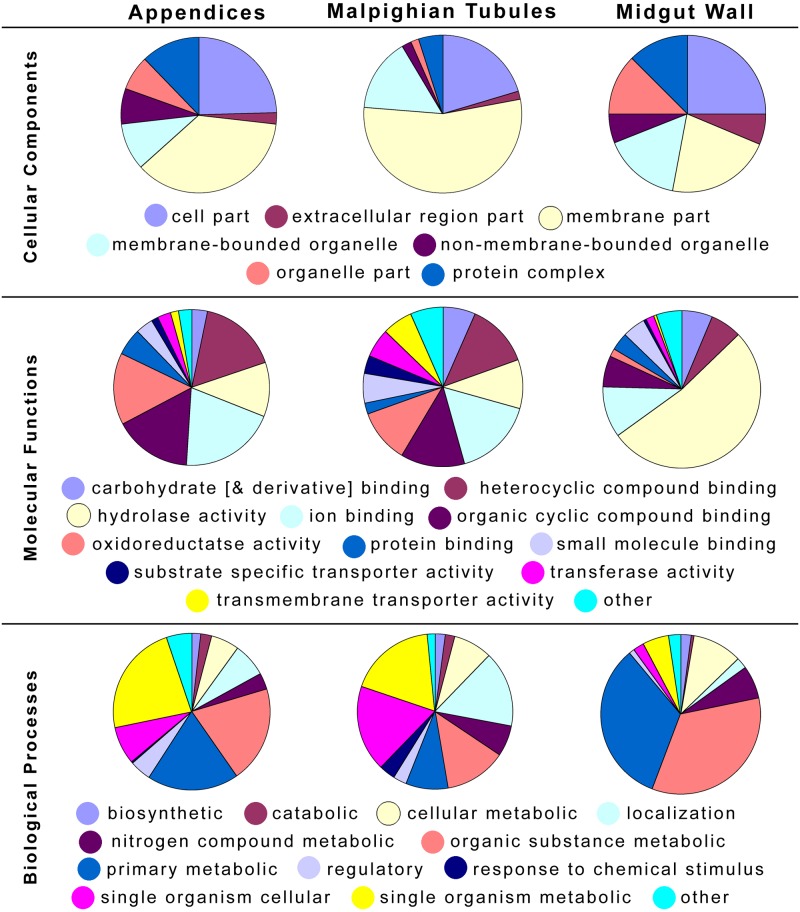
Pie charts of level 3 GO term categories for the differentially and highly expressed genes of the three tissue types.

Distribution of the gene ontology (GO) categories for only the both highly and differentially expressed transcripts of the three tissue types is summarized in Fig 3. Many of these transcripts in the midgut wall encoded for the aforementioned hydrolases. The Malpighian tubules transcripts were predominantly membrane or membrane-bound compounds. The differentially and highly expressed transcript profiles for the appendices are more similar to those of the Malpighian tubules to the midgut, suggesting similar function types despite the significantly different genes involved. The results of the KEGG [40] mapping of the most highly and differentially expressed genes per tissue are available in S2 Table and summarized as follows: Unique to the highly and differentially expressed genes in AoMs were enzymes involved in metabolism of glutathione (thioredoxin peroxidase, Enzyme Commission (EC) #1.11.1.15), alpha-linolenic acid (an omega-3 fatty acid) (EC 3.1.1.32) and glycerophospholipid (EC 1.1.1.8). Unique to the MpgTs were multiple enzymes used in sugar metabolism and antibiotic synthesis (EC’s 2.7.1.1, 4.1.2.13, 4.2.1.3, and 5.3.1.9), a retinol metabolism dehydrogenase (EC 1.1.1.105), and aminobenzoate degrading phosphatases (EC 3.1.3.2). Unique to the MGWall were psychosine hydrolases (EC 3.2.1.45), pectin depolymerase (EC 3.2.1.15), and alpha-fucosidase (EC 3.2.1.51). Both AoM and MGWall samples had differentially expressed chitodextrinases (EC 3.2.1.14) and hexosaminidases (EC 3.2.1.52).

### Specific protein types

Based on the BLASTx results and on past hypothesis for AoM function, I performed targeted searches for genes with specific functions, such as those involved in defense/multidrug resistance, biomineralization, and transport. I also performed a hidden Markov model (HMM) protein family domain search [41, 42] specifically for ATP-binding cassette (ABC) transporters, which are linked to xenobiotic elimination in Malpighian tubules [27] and which were hypothesized to be differentially expressed in Phasmatodea excretory organs [32]. The resulting lists of contigs are likely overestimates of the true gene number for each protein type, as some of these are likely alleles or variants of the same gene, or have their differences in the noncoding regions that our analysis software could not detect.

#### Multixenobiotic resistance and defense

I identified 187 ABC transporter / multidrug resistance protein contigs from several sub-families (S3 Table). Most of those with high expression (>100 RPKM) were expressed in the Malpighian tubules, and only one (Contig2190, an *ABCE1*) was also highly expressed in the other two. Several were significantly differentially expressed in certain tissues (Table 4), but none were both highly and differentially expressed. Of those BLASTx putatively identified to subfamily and member, two *ABCA1*’s and several *ABCG4*’s were differentially expressed in the AoMs. Both of those ABC transporters are cholesterol efflux transporters in other insects, known to work in concert to transport cholesterol and lipoproteins [43, 44]. Most of the other subfamilies either had differentially expressed representatives in multiple tissues or were broadly expressed. This includes several multidrug resistance proteins (MRPs), a class of the *ABCC* subfamily known as ATP-driven xenobiotic pumps [45].

**Table 4:** Differential expression of putative ATP-Binding Cassette (ABC) transporters in *Carausius* excretory tissues.

Category	Total	AoM	MpgT	MgWall	AoM + MpgT	MpgT + MGWall	AoM + MgWall
ABC[#]	2		1				
*ABCA1*	4	2					
*ABCA2*	1						1
*ABCA3*	13	4	2	4	1		
*ABCB10*	2						
*ABCB6*	2		1				
*ABCB7*	1						
*ABCB8*	2		1				
*ABCC11*	1						
*ABCC8*	1						
*ABCC*-MRP[#]	55	7	33	3	1		4
*ABCC-MRP1*	36	7	18	1	2		5
*ABCC-MRP3*	2	1		1			
*ABCC-MRP4*	10	1	3				3
*ABCC-MRP49*	7	3		3			1
*ABCC-MRP5*	7						
*ABCC*-*MRP7*	2	1					
*ABCD2*	1						
*ABCD3*	3						2
*ABCE1*	2						
*ABCF1*	1						
*ABCF2*	2						
*ABCF3*	1						
*ABCG1*	4					3	
*ABCG14*	1		1				
*ABCG20*	4		3				
*ABCG23*	3		2				
*ABCG4*	13	5	1	1		1	2
*ABCG5*	3		3				
*ABCG8*	1		1				

ABC transporters were identified with a profile hidden Markov model search [41] of the transcriptome with an ABC transporter PFAM domain query [42]. ABC transporter names are given with subfamily letter and subgroup number when identifiable. Values are the number of total sequences per subcategory in the transcriptome, and the number differentially expressed in each tissue or possible tissue pair. None of these sequences were highly expressed. For the full list, see S3 Table. AoM = Appendices of the Midgut, MGWall = Midgut Wall, MpgT = Malpighian tubules. MRP = multidtrug resistance protein.

**Fig 4:**
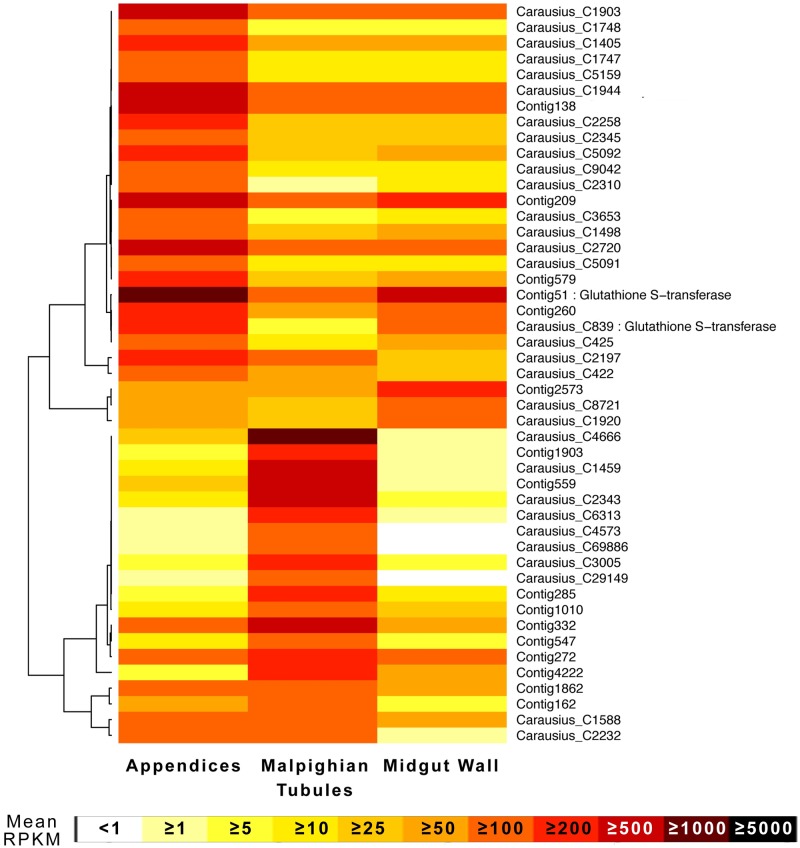
Expression heatmap for highly expressed, putative multixenobiotic detoxificaton genes in *Carausius* excretory tissue. All are cytochrome P450’s except the two labeled glutathione S-transferases [24]. Only genes with high expression in at least one tissue type (approximately >100 RPKM) were included. The original dendrogram, with branch lengths shortened for space, is provided, and the rows ordered accordingly.

I identified 181 other transcripts for proteins with noted functions in multidrug resistance / xenobiotic metabolism / defense. These included cytochrome p450s, glutathione-S-transferase, gram negative bacterial binding proteins (GNBBP), toll-related proteins, carboxylesterases, and one antimicrobial peptide (AMP) identified as having an attacin domain (Carausius_C61676). This is the first AMP identified in Phasmatodea, which I have named by convention for insect AMPs as Carausicin (Genbank Accession # KY271086), and which is differentially expressed in the MpgTs. Forty-seven defense genes, predominantly cytochrome p450s and gluthathione-s-transferases, were highly expressed in at least one tissue (Fig 4). One gluthathione-s-transferases (Contig51) was the 12^th^ most highly expressed gene in the AoMs. (S1 Table).

#### Fatty acid / Lipid and other transporters

I searched for any lipid metabolism genes based in part on Canavoso et al. [46]. This includes lipases and phospholipases, di- and triacylglycerol lipases, elongation of very long chain fatty acids” proteins, fatty-acid amide hydrolases and synthases, and fatty acid transporters. Of the 180 such transcripts identified, 46 were differentially expressed in the AoMs alone and another 30 in the AoMs and another tissue (Table 5). Some of these were among the most highly expressed transcripts of the AoMs, including several triacylglycerol lipases (Contig52, 8^th^; Carausius_C912, 15^th^) and fatty-acid binding proteins (Carausius_C392, 7^th^; Carausius_C197, 14^th^; Carausius_C198, 19th) (S1 Table). Several lipases were differentially expressed in the MGWall. All lipid phosphate phosphohydrolases and lipid storage droplets surface-binding proteins were differentially expressed in the AoMs, as well as several long-chain fatty acid transport proteins and phospholipases.

**Table 5:** Putative lipid/fatty acid/lipophilic substance metabolism/ and transport genes in *Carausius* excretory tissue.

Category	Total	# Differentially Expressed (# Highly Expressed)					
		AoM	MpgT	MgWall	AoM + MpgT	MpgT + MGWall	AoM + MGWall
AB-hydrolases	2	1 (1)		1			
Acidic Lipase	2	1 (2)		1			
Ca-Independent Phospholipase	2						
Diacylglycerol Lipase	3	2	1				
Elongation of VLCFAs Protein	9	2	1	1	1		1
Fat Storage-Inducing Transmembrane	1						
Fatty Acid Amide Hydrolase	8		3	1	1		
Fatty Acid Binding Protein	6	4 (3)	(3)	(3)	1		
Fatty Acid Hydroxylase	4	2	1				1
Fatty Acid Synthase	29	2			5		
Hormone-sensitive Lipase	1						
Lipase Maturation Factor	2	1					
Lipase, Other	4		1	1			
Lipid Export Permease	1						
Lipid Phosphate Phosphohydrolase	3	3 (1)					
Lipid Storage Droplets Surface-Binding	5	5 (2)					
Long-Chain Fatty Acid CoA Ligase	6	2 (1)	1		1		1
Long-Chain Fatty Acid Transport	9	1 (1)	4		2		
Lysophospholipase	3		2		1		
Lysophospholipid Acyltransferase	9	5					
Non-Specific Lipid-Transfer Protein	4	2					
Patatin-like Phospholipase	4	4					
Phospholipase Activator	3						
Phospholipase Inhibitor	1		1				
Phospholipase, Other	14	1 (1)	4 (2)	1	1		2
Phospholipid Hydroperoxide Glutathione Peroxidase	3	(3)	(3)	(1)	2		
Phospholipid Scramblase	2		1				1
Phospholipid-Transporting ATPase	14	4	1 (1)	1	3		
stAR-related Lipid Transfer Protein	3				1		
Triacylglycerol Lipase	23	4 (3)	1	7 (6)	2		3

Gene categories chosen in part based on Canavoso et al. [46]. Values are the number of total sequences per subcategory in the transcriptome, and the number differentially expressed in each tissue or possible tissue pair, with the number highly expressed in each tissue given in parentheses. VLCFA = very long chain fatty acid. AoM = Appendices of the Midgut, MGWall = Midgut Wall, MpgT = Malpighian tubules.

I expected to find lipophorins, as they are supposed to be the predominant lipid transport molecule in insects [46, 47]. However, BLASTx did not identify any. I then ran a profile HMM search for insect lipophorins as I did for ABC transporters, but still found none.

**Fig 5:**
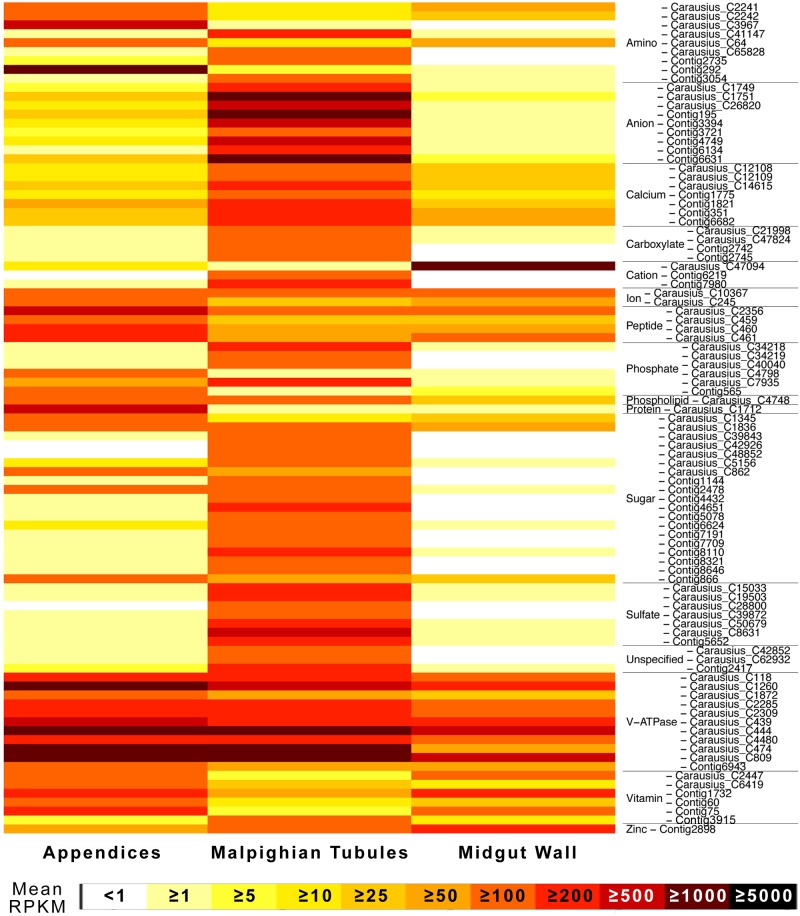
Expression heatmap for highly expressed, putative transporters in *Carausius* excretory tissue. Due to space constraints, only genes with high expression in at least one tissue type (approximately >100 RPKM) were included. Rows ordered alphabetically by gene function subcategory.

I identified 493 other putative transport protein genes. These included amino acid, anion, cation, Ca/Na/K, carboxylate, peptide, phosphate, phospholipid, sugar, sulfate, vitamin, water [aquaporins and aquaglyceroporins], and zinc transporters, plus 62 unspecified solute carriers. Of these, 93 were highly expressed with RPKMs >100 (Fig 5). Of those showing differential expression, most were over-expressed in the MpgTs. Zinc transporters were more expressed in the MGWall, while peptide and protein transporters more expressed in the AoMs. While some amino acid transporters were more expressed in the MpgT, others were very highly and differentially expressed in the AoMs, including one (Contig292), a sodium-dependent nutrient amino acid transporter and the 57^th^ most highly expressed transcript in the AoMs. Several V-ATPase transporters (proton pumps) were very highly expressed in the AoMs, some differentially, but most also highly in the other tissues as well. Many vitamin A transporters were expressed in the AoM and/or MGWall, while other vitamin transporters were predominantly in the MpgTs.

#### Biomineralization, calcium regulation, and phosphatases

**Fig 6:**
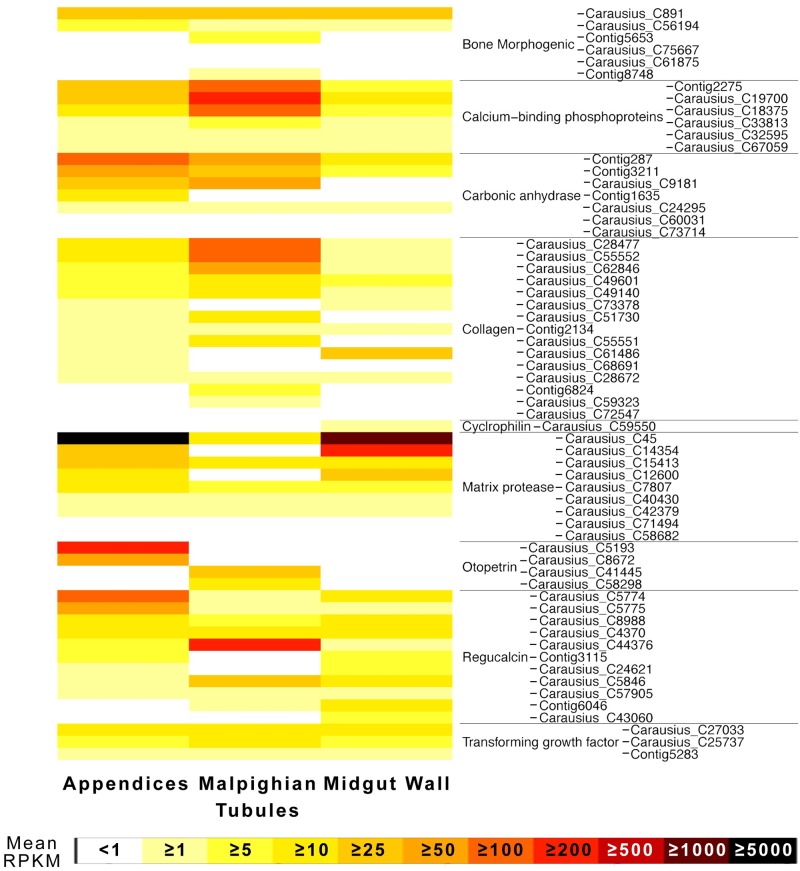
Expression heatmap for biomineralization genes in *Carausius* excretory tissue. Gene categories chosen in part based on Livingston et al. [48]. All identified biomineralization-type genes are included. Rows ordered alphabetically by gene function subcategory.

I searched for biomineralization genes based on the listing of Livingston et al. [48], due to the hypothesized existence in the AoMs of carbonic anhydrase [33] and otopetrin [49], and the known role of the Phasmatodea MpgTs in calcium elimination and deposition of unique calcium phosphate and/or calcium oxalate layers on the eggs [50–52]. These include bone morphogenic proteins, collagen, and regucalcin (one of which was highly and differentially expressed in the MpgTs, another in the AoMs), and matrix proteases (Fig 6). One of the latter (Carausius_C45) was identified as a collagenolytic serine protease, and was the third most highly expressed transcript in the AoMs and 27^th^ in the MGWall (S1 Table). I identified four otopetrin genes, two differentially expressed each in the AoMs and MpgTs, but only one (Carausius_C5193) highly expressed (325^th^ highest in AoM). Several calcium-binding phosphoproteins were differentially and highly expressed in the MpgT, while carbonic anhydrases were mostly expressed in the AoMs, though not highly.

A total of 318 contigs were identified as phosphatases. A few were highly expressed (S4 Table) nearly all in the MpgTs, including some of the most highly expressed of transcripts of the entire transcriptome: a lysosomal acid phosphatase (4^th^ most highly expressed MpgT contig and most highly expressed of all MpgT specific enzymes) and three prostatic acid phosphatase-like genes (6^th^, 8^th^, and 13^th^ most highly expressed MpgT contig). In contrast, alkaline and some protein phosphatases were more differentially expressed in the AoM, and nucleotide phosphatases in the MGWall.

## Discussion

The *de novo* transcriptome produced here provides the first clues to how the AoMs differ from the MpgTs on a molecular biology level. The data confirms what was known about MpgTs: they eliminate ions and other wastes from the hemolymph, and are actively involved in defense against and metabolism of xenobiotic and toxic compounds. The abundance of calcium-regulatory transcripts there matches what is known about Phasmatodea MpgTs and their relationship to calcium [50, 51]. The transcriptome also identified the known digestive enzymes in the midgut, including the Phasmatodea-specific pectinases [49], suggesting sufficient coverage. That the majority of identified transcripts had closest sequence homology to other Polyneoptera insect genes suggests low contamination and little chance that these are not endogenous *Carausius* genes.

The presence of phosphatases in the MpgTs is expected based on prior work with insects [53, 54], and their high abundance in Phasmatodea is likely because the order excretes excess calcium as a phosphate rather than as a carbonate as in most insects [50]. The highly expressed MpgTs alkaline phosphatases (S4 Table) thus likely function in calcium elimination. The highly expressed prostatic acid phosphatases, which in humans are produced by the prostate and are a serum tumor marker for prostate cancer, have many possible roles including excretion [54]. The possible use of Phasmatodea as models for human medical research is not unprecedented, as transcriptomes of *Drosophila* MpgTs promoted their use as models for human renal disease [21].

As predicted, the different tissues each express their own, unique set of genes involved in the transport, metabolism, and elimination of xenobiotics: ABC-transporters, glutathione-S-transferases, and cytochrome P450s (Table 4, Fig 4). The differential and often high expression of these proteins in the AoMs strongly suggests that the AoMs serve a function in multixenobiotic resistance, as hypothesized based on their physiology [32]. I do not reject the hypothesis that the observed differential staining patterns in Phasmatodea [32] can be due to these variations in xenobiotic compound transporters between AoMs and MpgTs, as has also been suggested by O’Donnell [1, 55]. The transcriptomes did not contain p-glycoproteins (*ABCB1*), a well-characterized xenobiotic pump in insects [1], but did contain an antimicrobial peptide with an attacin domain [56]. I am working towards identifying further antimicrobial peptides in the Phasmatodea.

Notably, I reject the hypotheses that the AoMs are involved in ion homeostasis, including the cation hypothesis from Shelomi & Kimsey [32], based on their relative lack of such transporters. Instead, the AoMs differentially and highly express peptide and protein transporters and phosphatases, and several V-type proton ATPases (Fig 5) that may or may not be involved in xenobiotic compound transport. I cannot rule out a function for the AoMs in peptide homeostasis. V-ATPases are known to be responsible for normal formation of uric acid concretions and calculi in insect MpgTs [57], and may explain the concretions observed in *Peruphasma schultei* AoMs [30]. Otherwise, they are critical to the transport function of Malpighian tubules [55, 58], and likely drive the transport in the AoMs as well. Although the AoMs seem to differentially absorb cationic stains [32], they did not differentially express cation transporters, suggesting that those stains were picked up through the action of other transporter types: the ABC transporters and/or an unidentified pump driven by the electric potential generated by V-ATPases.

The differential and often high expression of lipid- and fatty acid metabolism, binding-, and transport genes in the AoMs (Table 5), including cholesterol and lipoprotein specific ABC transporters (Table 4) [43, 44], suggests a key role for these organs in movement and homeostasis of lipids, lipophilic substances (including fat soluble vitamins), and/or lipoproteins. This is a completely novel hypothesis for AoM function. The known yellow color of the AoM ampule contents and the lipophilic nature of the *P*. *schultei* concretion [30] further suggest that the AoMs may contain a nonpolar fluid or at least be well suited to the transport of lipophilic substances. However, the AoMs are known to pick up water-soluble stains with ease [32]. The BLASTx and *nhmmr* results did not show lipophorins in any tissues, despite their key role in lipid transport in insects [46, 59], but did find di- and triacylglycerol transporters [47]. *Carausius* may express lipophorins in the hemolymph, which would have been washed away in our dissections and not sampled. Alternatively, some or all Phasmatodea use a different lipid transport system than generally known in insects, which warrants further study. Vitellogenins are another possible insect lipoprotein [59], and I found some differentially but not highly expressed in the MGWalls (S1 Table). de Sinéty [60] and Savage [52] reported that certain Phamsatodea calciferous Malpighian tubules terminate in the fat body (cells of Sidorot), but neither de Sinéty nor Shelomi et al. [30] found evidence that the AoMs terminate at any tissues. They may nonetheless interact with fat bodies and pass near them, transporting lipophilic substances to and/or from the midgut lumen and fat body or hemolymph, possibly having evolved this ability in absence of lipophorins.

Certain insect lipoproteins in the hemolymph are known to bind to xenobiotics or enable coagulation of the hemolymph following microbial infection [59], so the AoM lipid-related genes may thus also be defensive. Lipid droplets in *Drosophila* are known to serve as protein storage depots as well [61]. The combination of lipid storage droplet binding sites and peptide transporters among the differentially and highly expressed AoM genes, including a highly expressed sodium-dependent nutrient amino acid transporter, would thus suggest a role of the AoMs in transporting lipid-droplet bound proteins and peptides. Lastly, the high and/or differential expression of a retinol (a fat soluble vitamin) dehydrogenase (Table 3) and several alpha tocopherol (a fat soluble vitamin) transfer proteins (S1 Table) suggests the AoMs may function in the transport of fat-soluble vitamins as well as nutrient amino acids. Ultimately the literature on fat metabolism and transport in insects is incomplete [46,47], though perhaps the Phasmatodea have just proven their worth as model insects for further study of this field.

The presence of beta-actins in the AoMs is expected given the high motility of the tubules. The actins are likely involved in the harmonic motion of the AoM tubules in the hemolymph. I also absolutely confirm the predictions of Monteiro et al. [33] that the AoMs express carbonic anhydrase (Fig 6), which alkalinizes the gut lumen. The effects of this alkalinization could be to counteract or disable midgut digestive enzymes, but it may also have functions within the AoM tubules and ampules themselves. Once again identified in the AoMs [49] is high and differential expression of the otolith formation and biomineralization gene, otopetrin [62] (Fig 6). The two otopetrins differentially expressed in the AoMs (Carausius_C5193 & Carausius_C8672) have the 1^st^ and 7th greatest log2 fold change recorded between the AoM and MpgT (S1 Table). Only Carausius_C5193 is highly expressed [mean RPKM of 274.4 in the AoMs]. In insects, otopetrin is highly expressed in glowworm light organs along with carbonic anhydrase and several defensive compounds [63], and otopetrin is upregulated in mosquitoes after topical juvenile hormone application [64]. More generally, otopetrin is known to regulate calcium homeostasis [62], but as the calcium transport genes were predominantly expressed in the MpgTs, I still cannot explain what this gene does in the AoMs. In mice, otopetrin is part of a signaling pathway in adipose tissue that reduces obesity [65], so it is possible that Phasmatodea AoM otopetrin are part of the lipid metabolism/transport functionality rather than biomineralization. Future tests using RNAi or other knockdown methods targeting otopetrin could answer this question, but RNAi has not yet been attempted in a Phasmatodea.

## Conclusion

By combining prior physiological assays of the Phasmatodea-specific midgut appendices with the new transcriptomic data, I can provide the first conclusions (Table 6) for what these enigmatic organs do since their earliest recorded description over a century ago. The main functions of the appendices include, but are not limited to: 1) Multixenobiotic resistance by metabolizing and transporting such compounds out of the hemolymph, targeting different compounds from the Malpighian tubules. 2) Transport and homeostasis of lipids and fatty acids, lipoproteins, and/or lipophilic substances such as nutrients, xenobiotics, and lipid storage droplet-bound proteins, possibly in absence of lipophorin. 3) Contributing to the alkalization of the posterior midgut by carbonic anhydrase activity rather than cation excretion.

**Table 6:** First conclusions for the functions of the Phasmatodea appendices of the midgut and supporting evidence.

Function of the Appendices	Evidence (•) and Hypothesized Links (*)	Source
1. Multixenobiotic resistance (metabolism, excretion, and defense against foreign compounds, toxins, microbes)	• Elimination of injected stains unlike those of Malpighian tubules	Shelomi & Kimsey [32]
	• Differential expression of *ABCC* xenbiotic export pumps (Multidrug resistance proteins)	Table 4, S3 Table
	• High and differential expression of cytochrome P450s and glutathione S-transferases (metabolism and detoxification genes)	Fig 4
	* High and differential expression of V-type proton ATPase (transport gene), but not of waste/homeostasis transporters	Fig 5
2. Lipophilic compound (nutrients, lipoproteins, xenobiotics) transport and regulation	• High and differential expression of fatty acid binding proteins, lipid storage droplet binding proteins, triaglycerol lipases, and fat-soluble vitamin transfer genes	Table 3
	• High and differential expression of alpha-linolenic acid and glycerophospholipid metabolism enzymes (based on KEGG mapping)	S2 Table
	• Differential expression of cholesterol and lipoprotein transporting ABC transporters	Table 4, S3 Table
	• Differential expression of lipid phosphate phosphohydrolases, long-chain fatty acid transport proteins, and a number of other lipid-related genes.	Table 5
	• High and differential expression of (fat soluble) vitamin A and vitamin E transporters	Table 3, Fig 5
	* Absence of lipophorins demands alternative transport method	-
3. Alkalization of the posterior midgut via biomineralization proteins	• pH of midgut reaches or passes neutral at the start of the Appendices	Shelomi et al. [30], Monteiro et al. [33]
	• High carbonic anhydrase activity measured	Moneteiro et al. [33]
	• Differential expression of carbonic anhydrase, but not cation transporters	Figs 5 and 6
	* Differential expression of calcium-carbonate related gene Otopetrin	Fig 6

## Materials and methods

### Insect dissection and RNA extraction

Adult *Carausius morosus* females were obtained from private cultures maintained on blackberry (*Rubus* sp.) leaves. Three biological replicates of three insects each were prepared. Tissue removal was performed on ice with tools sterilized in ethanol and treated with RNaseZap. Insects had their heads and last abdominal segments removed with scissors and the entire gut pulled out using watchmaker’s forceps and placed into a Petri dish of 70% ethanol. If necessary, a full-length, lateral incision was made with Castroviejo scissors and the entire digestive tract removed. Gut contents within their peritrophic membrane could often be pulled from the entire midgut via the anterior opening. Incisions were made at the beginning and end of the posterior midgut [defined as the region studded with appendix ampules], taking care not to cut through the appendix tubules, and just before and after the origin of the Malpighian tubules. The section of midgut between these, the “post-posterior midgut” [30], was used as the midgut wall. Tissue rings were washed in a separate dish of ethanol to remove contaminants, with care to ensure all appendix tubules were removed from among the Malpighian tubules. Tissues (approximately 20μg each) were then immediately placed into Invitrogen^™^ RNAlater^™^ Stabilization Solution (ThermoFischer) and macerated in a frozen Tissue Lyser with metal beads. RNA was extracted with an innuPREP RNA MiniKit (Analytik-Jena AG) and purified with theRNeasy^®^ MinElute^®^ cleaning kit (Qiagen^®^) following the manufacturers’ protocols. RNA quality was tested with an Experion^™^ RNA chip (Bio-Rad) on an Agilent 2100 Bioanalyzer following the manufacturers’ protocols.

### Sequencing, de novo transcriptome assembly

The three replicates of three tissues each were sent over dry ice to the Max Planck-Genome-centre, Cologne, Germany (http://mpgc.mpipz.mpg.de/home/) for RNA library production including polyA enrichment with an Illumina HiSeq 2500 Sequencer with 100bp paired-end reads, followed by raw data processing, data quality filtering, and data de-multiplexing. This generated 20 million reads (4.00 gigabases) of data per library.

The libraries were quality tested, trimmed (minimum contig length 250bp), and pooled for assembly of a *de novo* transcriptome using CLC Genomics Workbench v8.0 (Qiagen^®^). This transcriptome was tested for duplications, revealing 64,323 singlets and 20,683 contigs forming an overlap or contained within another. The resulting contigs were fused using CAP3 [38] with standard parameters. This assembled transcriptome of 73,143 contigs made up of approximately 70 Mbp is available on Dryad.

### RNA-Seq and data analysis

RNA-Seq analysis, which has been successfully used in Phasmatodea before [49, 66], was performed by the Max Planck-Genome-centre with the Cufflinks v2.2.1 suite of tools (http://cole-trapnell-lab.github.io/cufflinks/) by mapping the original library reads to the CAP3 *de novo* transcriptome with TopHat, assembling with Cufflinks, merging with Cuffmerge, and calculating differential expression between each possible pair of tissue types with Cuffdiff [67]. The expression values were calculated as RPKM (reads per kilo base per million mapped reads), and the mean RPKM for each tissue type and gene is available in S1 Table. Contigs were tentatively identified via BLAST using the CLC Genomics Workbench v8.0.

I identified contigs that Cufflinks analysis demonstrated were statistically significantly (p<0.05) differentially highly expressed in one tissue type compared to the other two. These are labeled in S1 Table and were matched to transcriptome sequences using the online bioinformatics software Galaxy version 1.0.2 to manipulate the data and produce a fasta file. This was further annotated via Blast2GO v3.0.11 [39] using BLASTx (e-value<1e^-10^) to search against the GenBank non-redundant protein database. Transcripts were also scanned for conserved protein domains via an InterProScan and mapped and annotated within Blast2GO.

### Functional domain search for specific gene types

To search for transcripts with ABC transporter or lipophilin domains, I obtained insect query sequences for such proteins using the PFAM database (http://pfam.xfam.org/) [42], eliminating putative and uncharacterized proteins. These were aligned with MUSCLE v3.3 [68], converted to Stockholm format (.sto), and backtranslated into a nucleotide.sto file with EMBOSS *Backtranambig* software (http://www.ebi.ac.uk/Tools/st/emboss_backtranambig/), and converted into a pHMM query file with the *hmmbuild* package of HMMER v3.1. This file was used to query the transcriptome with *nhmmer* [41]. These identified contigs were matched to their expression data from the RNA-Seq dataset. To make heatmaps I used the *heatmap3* package for R v3.3.1 [69].

## Supporting information

S1 Fig Digestive and excretory system of *Carausius morosus*. A) Schematic and B) dissection of the alimentary canal from *Carausius morosus*, typical of other Phasmatodea [32]. The gut is presented unstained, so tubules are not visible to the naked eye. The appendices appear on the posterior midgut. The Malpighian tubules originate at the midgut/hindgut junction, trailing over the posterior midgut before going towards the posterior end of the insect. The gut section between the two, the “post-posterior midgut,” was used for our midgut wall (MGWall) samples, excluding any tubules. Key: AMG = anterior midgut. AoM = appendices of the midgut. MMG = middle midgut. MpgT = Malpighian tubules. PMG = posterior midgut. PPMG = post-posterior midgut.
(TIFF)

S2 Fig Top-Hit species distribution for BLASTx results of the *Carausius* excretory tissue *de novo* transcriptome assembly. (TIFF)

S1 Table Descriptions and differential expression statistics for *C*. *morosus* excretory tissue transcripts. (XLS) Putative descriptions based on BLASTx results. RKPM = Mean reads per kilobase per million mapped reads. Contigs are “highly expressed” in their tissue type if the RPKM is greater than 10x the mean. For each tissue type pair, the log_2_ fold change in expression based on Cufflinks analysis is given, with negative values indicating higher expression in the first tissue type of the pair as ordered in the heading. “Differential expression” is based on significantly (p<0.05 based on Cufflinks analysis of the test statistic) higher differential expression in the tissues as follows: AoM = Appendices of the Midgut, excretory = AoM & MpgT, MGWall = Midgut Wall, midgut = AoM & MGwall, MpgT = Malpighian tubules, no = no differential expression, rear = MpgT+MGWall, zero = no significant expression in any tissue type.
(XLS)

S2 Table Distribution of KEGG pathways of the highly and differentially expressed transcripts of the *C*. *morosus* excretory tissue transcripts. (XLS)

S3 Table *C*. *morosus* transcriptome contigs identified as ATP-binding cassette (ABC) transporters. ABC transporters were identified with a profile hidden Markov model search [41] of the transcriptome with an ABC transporter PFAM domain query [42]. ABC transporter names are given with subfamily letter and subgroup number when identifiable. “Differential expression” is based on significantly (p<0.05 based on Cufflinks analysis of the test statistic) higher differential expression in the tissues as follows: AoM = Appendices of the Midgut, excretory = AoM & MpgT, MGWall = Midgut Wall, midgut = AoM & MGwall, MpgT = Malpighian tubules, no = no differential expression, rear = MpgT+MGWall, zero = no significant expression in any tissue type.
(XLS)

S4 Table Putative phosphatase genes in *Carausius* excretory tissue. “Differential expression” is based on significantly (p<0.05 based on Cufflinks analysis of the test statistic) higher differential expression in the tissues as follows: AoM = Appendices of the Midgut, excretory = AoM & MpgT, MGWall = Midgut Wall, midgut = AoM & MGwall, MpgT = Malpighian tubules, no = no differential expression, rear = MpgT+MGWall, zero = no significant expression in any tissue type.
(XLS)
