# Supplementary figures and images for: De novo transcriptome analysis of the excretory tubules of Carausius morosus (Phasmatodea) and possible functions of the midgut ‘appendices’
Source: PLoS One. 2017 Apr 6;12(4):e0174984. doi: 10.1371/journal.pone.0174984 (PMC5383107; doi:10.1371/journal.pone.0174984)

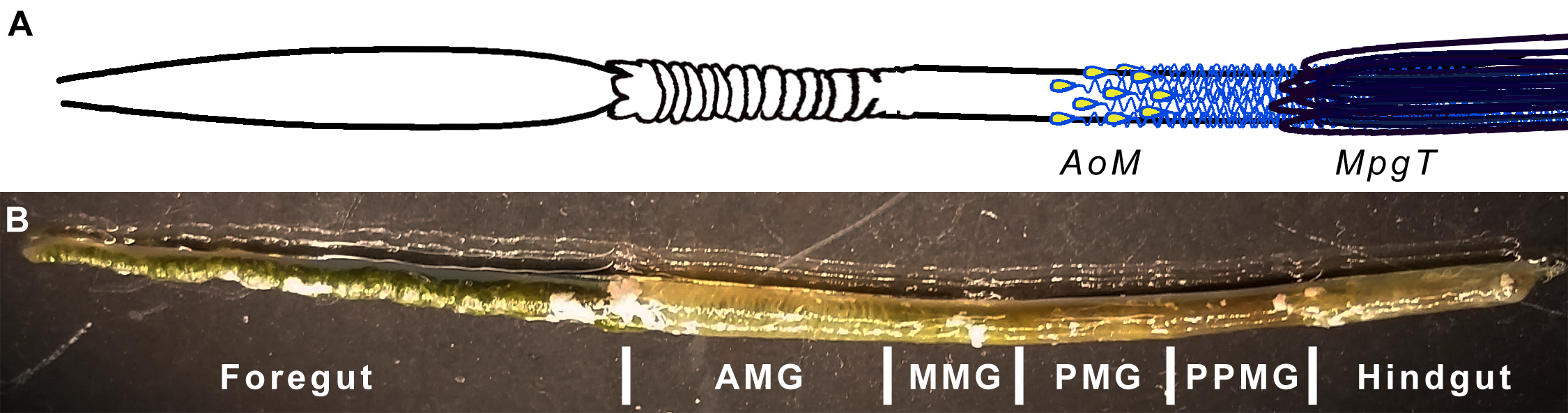

Supplement: S1 Fig — A) Schematic and B) dissection of the alimentary canal from Carausius morosus, typical of other Phasmatodea [32]. The gut is presented unstained, so tubules are not visible to the naked eye. The appendices appear on the posterior midgut. The Malpighian tubules originate at the midgut/hindgut junction, trailing over the posterior midgut before going towards the posterior end of the insect. The gut section between the two, the “post-posterior midgut,” was used for our midgut wall (MGWall) samples, excluding any tubules. Key: AMG = anterior midgut. AoM = appendices of the midgut. MMG = middle midgut. MpgT = Malpighian tubules. PMG = posterior midgut. PPMG = post-posterior midgut. (TIFF) [file pone.0174984.s001.tiff]

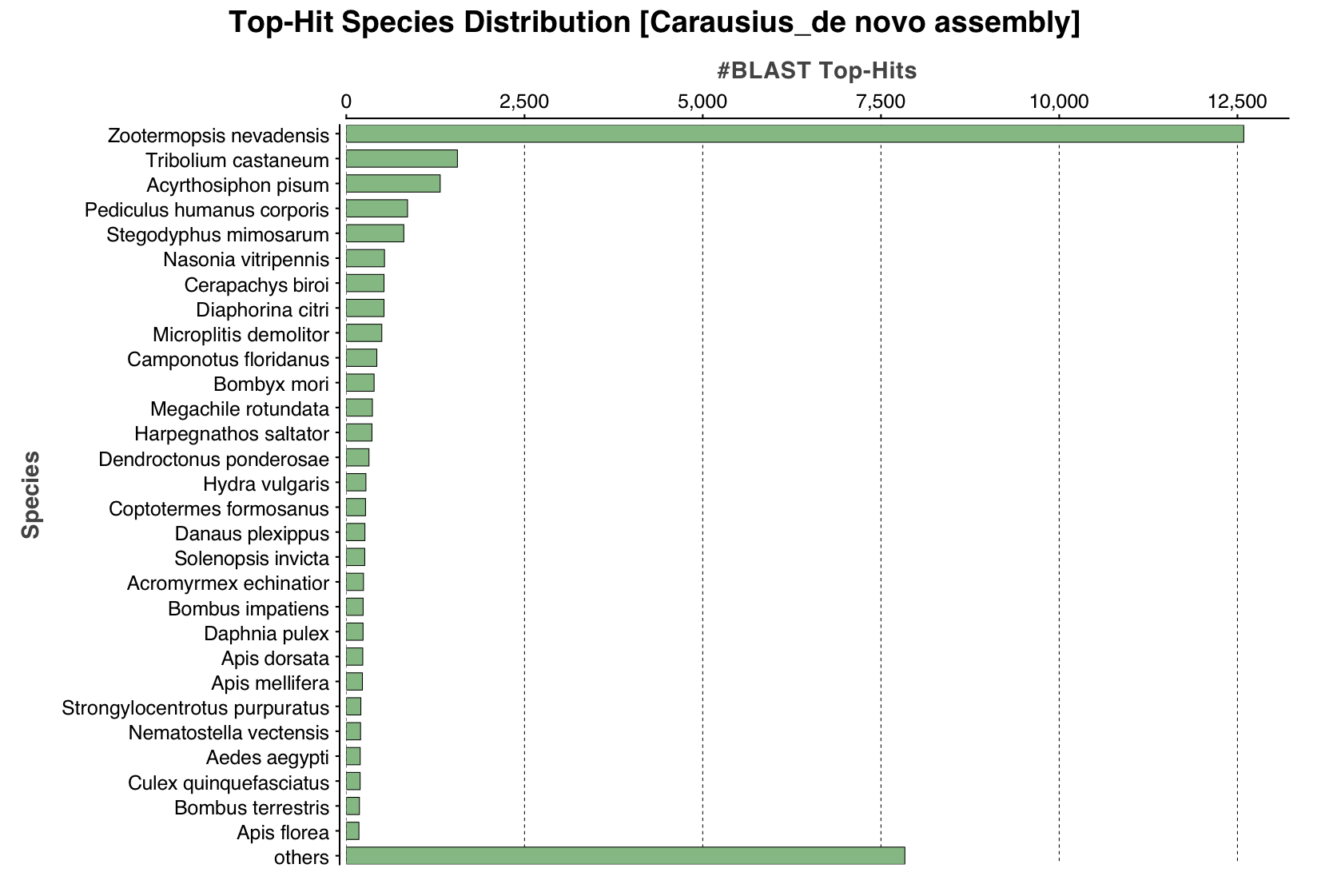

Supplement: S2 Fig — (TIFF) [file pone.0174984.s002.tiff]
